# Molecular alterations in apoptotic pathways after PKB/Akt-mediated chemoresistance in NCI H460 cells

**DOI:** 10.1038/sj.bjc.6601876

**Published:** 2004-05-11

**Authors:** S Hövelmann, T L Beckers, M Schmidt

**Affiliations:** 1 ASTA Medica Oncology, Weismüllerstr. 45, D-60314 Frankfurt, Germany

**Keywords:** protein kinase B/Akt, apoptosis, chemoresistance

## Abstract

Protein kinase B/Akt has been described as a central mediator of antiapoptotic signals in cancer cells. Furthermore, Akt has been shown to affect cell cycle progression and proliferative pathways and to possess a potential function in tumorigenesis and chemoresistance. In this study, we show that the ectopic expression of a constitutively active form of Akt1 (CA-Akt1) results in enhanced chemoresistance of NCI H460 human NSCLC cells towards a panel of chemotherapeutic agents. To understand the molecular alterations leading to impaired chemosensitivity mediated by activated Akt, we analysed various apoptotic pathways, including the activation of p53, caspases 3, 7, 8, and 9, release of cytochrome *c* from mitochondria, and the expression levels of pro- and antiapoptotic proteins such as Bcl-2, Bcl-x_L_, Bcl-x_s_, Bax, or Bfl-1. We observed that expression of CA-Akt did not interfere with single defined apoptotic switches, but modulated the apoptotic threshold of several apoptotic pathways towards increasing the threshold of onset. In particular, we found that CA-Akt-expressing cells displayed increased expression of the antiapoptotic Bcl-2 family member protein Bcl-x_l_, and a delayed onset of the p53 pathway after treatment with cisplatin or Mitoxantrone. Thus, our data suggest that Akt mediates chemoresistance in NHI H460 cells by interfering with and delaying the onset of various apoptotic pathways. A complete inactivation of apoptotic pathways was observed in none of the molecular alterations investigated. Our data strengthen the role of Akt as a central mediator of cell survival signals and/or chemoresistance and as an attractive target for cancer cell chemosensitisation.

The process of malignant transformation requires cells to undergo a variety of molecular alterations to acquire growth properties that allow them to divide indefinitely and in an uncontrolled fashion under conditions of low oxygen and poor nutrient supply. Such conditions would normally induce programmed cell death, and malignant cells additionally have to acquire molecular mechanisms to evade apoptotic stimuli in order to survive and to form solid tumours. It is conceivable that antiapoptotic signalling cascades play an equally important role in the development of the pathological cancer phenotype as the dysregulation of proliferative signalling cascades.

Phosphatidyl-inositol 3-kinase (PI3 K), which relays various signalling pathways via the production of phosphatidyl-inositide lipids, has been identified as a central mediator of cell growth, metabolism, migration, and survival (reviewed in Wymann *et al*, 2003). PI3 K regulates cellular functions by recruiting PI(3, 4, 5)P_3_ binding proteins to the plasma membrane preferentially via pH domains. The prototype of these molecules is protein kinase B/Akt. Binding to PI(3, 4, 5)P_3_ results in a conformational change which makes Akt susceptible to phosphorylation at threonine 308 and serine 473 (Alessi and Cohen, 1998). Phosphorylation of Akt results in its activation and dissociation from the membrane. The resolution of the crystal structures of activated Akt and inactive Akt2 has provided further clues about their regulation and activation (Yang *et al*, 2002; Huang *et al*, 2003). Substrates of Akt are phosphorylated within the consensus sequence RXRXXS/T (Obata *et al*, 2000). Several target proteins of Akt involved in antiapoptotic signalling, the regulation of the cell cycle, and insulin homeostasis have been described.

GSK3 was the first protein known to be inactivated by Akt via phosphorylation at serine 9 (Cross *et al*, 1995). Besides the regulation of glycogen synthesis, GSK3 regulates several intracellular signalling pathways including AP1 (Scalia *et al*, 2001), CREB (Cross *et al*, 1995), and the tumour-suppressor gene product APC (Zumbrunn *et al*, 2001). Akt can promote cellular survival by inhibiting proteins involved in the regulation of apoptosis. One such mechanism is the phosphorylation of the proapoptotic protein Bad at serine 136 by Akt, which creates a binding site for 14-3-3 proteins and thereby prevents Bad from binding and inhibiting the antiapoptotic protein Bcl-x_L_ (Datta *et al*, 1997). Another direct mechanism is the Akt-mediated phosphorylation of caspase 9 at serine 196 inhibiting its protease- and apoptosis-initiating activity (Cardone *et al*, 1998). c-FLIP, a dominant-negative homologue of caspase 8, is upregulated in tumour cell lines on a transcriptional level by the Akt pathway through a yet unknown mechanism (Panka *et al*, 2001). The apoptosis signal-regulating kinase 1 (Ask1) is phosphorylated by Akt at serine 83, leading to its inhibition and a reduced activation of JNK, which under certain circumstances can promote apoptosis (Kim *et al*, 2001). The forkhead transcription factor FKHRL1 is phosphorylated by Akt at threonine 32 and serine 253, resulting in its retention in the cytoplasm by 14-3-3 proteins. Thus, FKHRL1-mediated transcription of proapoptotic proteins such as the Fas ligand is reduced and cell survival is promoted (Brunet *et al*, 1999). Other substrates of Akt include phosphofructokinase 2 (Deprez *et al*, 1997), I*κ*-B kinase from the NF-κB pathway (Ozes *et al*, 1999), and endothelial NO synthase (Dimmeler *et al*, 1999).

Akt can influence proliferative and cell cycle regulatory pathways and thereby indirectly modulate tumour chemosensitivity by the regulation of Cyclin D stability (Muise-Helmericks *et al*, 1998) and inhibition of p27^Kip1^ protein levels (Collado *et al*, 2000). Furthermore, Akt has been shown to directly phosphorylate the cyclin-dependent kinase inhibitors p21^WAF1^ at threonine 145 and serine 146 (Zhou *et al*, 2001) and p27^Kip1^ at threonine 157 (Liang *et al*, 2002), leading to their cytosolic retention and neutralisation of their growth-inhibitory effect. In addition, cytoplasmic p21^WAF1^ binds to ASK1-inhibiting apoptosis (Asada *et al*, 1999). The p53 pathway is influenced by Akt through phosphorylation of the p53-regulating protein MDM2 at residues serine 166 and 186 (Mayo and Donner, 2001). Akt-mediated phosphorylation of MDM2 results in the translocation of MDM2 from the cytoplasm into the nucleus, leading to reduced cellular p53 protein levels and decreased p53 transcriptional activity.

Since PKB/Akt is involved in such a multitude of apoptosis regulatory pathways, it is not surprising that Akt is overexpressed in a variety of human tumour cell lines and cancers (Bellacosa *et al*, 1995; Ringel *et al*, 2001; Roy *et al*, 2002) and a mediator of oestrogen resistance in breast cancer cells (Campbell *et al*, 2001). Akt activity in cancers can either be deregulated by constitutive activation or by mutation of PTEN/MMAC1, a phosphatase that directly counteracts Akt through the dephosphorylation of PI-3-, 4-, and 5-P_3_ (Stambolic *et al*, 1998). Besides being overexpressed or constitutively activated in certain types of cancer, Akt has been implicated in modulating sensitivity of cancer cells towards standard chemotherapy (Hayakawa *et al*, 2000; Page *et al*, 2000; Schmidt *et al*, 2002; Knuefermann *et al*, 2003).

Although many apoptotic pathways modulated by Akt have been described, relatively little is known about the actual molecular mechanisms by which Akt suppresses apoptosis after chemotherapeutic treatment of cancer cells. To establish a cellular system that allows in-depth analysis of these pathways, we transfected NCI H460 human NSCLC cells with an expression vector encoding a constitutive form of Akt1 (Schmidt *et al*, 2002). Here we describe the effects of CA-Akt1 on the modulation of chemosensitivity towards several chemotherapeutic regimen in stable cell clones of NCI H460 NSCLC cells. Furthermore, we describe molecular alterations in various apoptotic pathways that may account for the desensitising effects observed in CA-Akt1-expressing NCI H460 cells.

## MATERIALS AND METHODS

### Materials

Anti-Akt, anti-phospho-Akt, anti-GSK, anti-PARP, and anti-caspase antibodies were obtained from Cell Signalling Technology, Inc. (Beverly, MA, USA); anti-Bcl2, anti-Bcl-x_s/l_, anti-A1/Bfl-1, anti-Bax, and anti-p53 antibodies were purchased from Santa Cruz Biotechnology, Inc. (Santa Cruz, CA, USA); anti-MDM2 antibody was obtained from Oncogene Research Products (Boston, MA, USA); M2 anti-FLAG antibody, *β*-actin antibody, and all other reagents were purchased from Sigma Chemical (St Louis, MO, USA) unless otherwise specified.

### Cells and cell culture

NCI H460 human lung cancer cells were obtained from ATCC and maintained in RPMI 1640 medium supplemented with 10% FBS, 2 mM L-glutamin, 100 u ml^−1^ penicillin G, and 100 *μ*g ml^−1^ streptomycin.

### Establishment of farnesylated Akt1 expression clones in NCI H460 cells

NCI H460 cells were transfected with pcDNA3.1. Hygro vector containing farnesylated Akt1 devoid of the PH domain described earlier (Schmidt *et al*, 2002). Transfection was performed with the Fugene-6 transfection kit (Roche Diagnostics, Indianapolis, IN, USA) according to the manufacturer's recommendations. Stable NCI H460 clones were selected in medium containing 50 *μ*g ml^−1^ Hygromycin B (Roche Diagnostics, Indianapolis, IN, USA). Stable clones were analysed for expression of ectopic FLAG-tagged Akt1 devoid of its PH domain and containing the farnesylation sequence by immunoblotting with the M2 antibody specific for the FLAG tag. Clones that were both, resistant towards the respective antibiotic and positive on protein level, were selected for further studies. Clones expressing farnesylated Akt1 were named NCI H460-Akt1. Experiments were conducted with at least two different clones.

### Cytotoxicity assay

Cells were seeded in 100 *μ*l medium supplemented with 0.5% FCS into 96-well culture plates. After 24 h incubation, cells were treated with various doses of chemotherapeutic drugs as indicated in 50 *μ*l volume. At 72 h after addition of the compounds, cell viability was assayed by adding 75 *μ*l of 1 mg ml^−1^ sodium 3′-[1-(phenyl amino carbonyl-3,4-tetrazolium]-bis (4-methoxy-6-nitro-) benzene sulphonic acid (XTT) in RPMI containing 7.66 *μ*g ml^−1^ *N*-methyl dibenzopyrazin methyl sulphate (PMS). After incubating the cells for 3 h at 37°C in a CO_2_ incubator, cell viability was determined by measuring the optical absorbance at a wavelength of 490 nm and normalization with the corresponding control cells. Each concentration was assayed in quadruplicates and the assay for each cytostatic was independently repeated at least three times.

### Immunoblot analysis

Cells were lysed in a buffer containing 50 mM Tris, pH 7.4, 150 mM NaCl, 1% NP-40, 50 mM NaF, 1 mM Na_3_VO_4_, and 1 mM phenylmethylsulphonyl fluoride. Lysates were cleared by centrifugation and supernatants were collected. Equal amounts of lysate protein were used for Western blot analysis with antibodies as indicated. Specific signals were visualised by use of the ECL chemoluminescence detection kit (Amersham, Braunschweig, Germany).

### Determination of Akt1 phosphorylation

Cells were seeded onto six-well plates in medium containing 0.5% serum. After starvation overnight, cells were stimulated for 15 min with medium containing 10% serum or medium additionally supplemented with a cocktail containing each 10 ng ml^−1^ of EGF, PDGF, and IGF-1. Cell lysates were then analysed in immunoblot assays with antibodies specific for phospho-Akt (Thr 308) or phospho-Akt (Ser 473). Signals were visualised by use of the ECL chemoluminescence detection kit (Amersham, Braunschweig, Germany).

### Akt kinase assay

The Akt kinase assay was performed using the Akt Kinase Assay Kit from Cell Signalling Technology, Inc. with GSK-3 fusion protein as substrate. Cells were seeded out in 10 cm dishes and switched to medium containing 0.5% serum after attachment. After starvation overnight, cells were either kept unstimulated or were stimulated for 20 min with medium containing 10% FCS supplemented with a cocktail containing each 10 ng ml^−1^ of EGF, PDGF, and IGF-1. Cells were lysed and lysates clarified by centrifugation. Supernatants were swirled for 3 h at 4°C with an anti-Akt-agarose conjugate. After three times washing with lysis buffer and with kinase buffer, the immunoprecipitates were subjected to an *in vitro* kinase reaction with 40 *μ*l of reaction mixture containing kinase reaction buffer supplemented with 200 *μ*M ATP and 1 *μ*g GSK-3-fusion protein. The reaction was allowed to process at 30°C for 30 min and stopped by boiling the samples in SDS sample buffer for 5 min. Immunoprecipitates were separated by 12.5% SDS–PAGE and then analysed in immunoblot assays with antibodies specific for phospho-GSK (Ser 21/9) and Akt. Signals were visualised with the ECL chemoluminescence detection kit (Amersham, Braunschweig, Germany).

### Colorimetric detection of caspase activation

The activation of the initiator caspases 8 and 9, as well as the effector caspase 3, was determined by *in vitro* assays with cell lysates using the caspase 3 cellular activity assay kit, the caspase 8 assay kit (both Calbiochem-Novabiochem, San Diego, CA, USA), and the caspase 9 colorimetric assay kit (R&D Systems, Minneapolis, MN, USA). Cells were seeded out in 10-cm dishes and switched to medium containing 0.5% serum after attachment. After starvation overnight, cells were treated with 400 nM Mitoxantrone or 20 *μ*M cisplatin for 24 and 36 h, respectively. Cells were lysed and lysates were clarified by centrifugation. Supernatants were subjected to caspase activity assays according to the manufacturer's recommendations with specific peptide substrates (DEVD-pNA for caspase 3, Ac-IETD-pNA for caspase 8, and LEHD-pNA for caspase 9). Active caspases lead to the proteolytic cleavage of the peptide substrates and thereby the release of *p*-nitroanilin, which was quantified with the Victor Multilabel Counter at a wave length of 405 nm.

### Fractional centrifugation and cytochrome *c* ELISA

For analysing the distribution of cytochrome *c* between mitochondria and cytosol cells were fractionally centrifuged and a cytochrome *c* ELISA (EMD Biosciences, Inc., San Diego, CA, USA) was performed. Cells were seeded out in 10-cm dishes and switched to medium containing 0.5% serum after attachment. After starvation overnight, cells were treated with 400 nM Mitoxantrone or 20 *μ*M cisplatin for 8 h. After treatment, cells were removed from the dish, resuspended in buffer A (20 mM Hepes-KOH, pH 7.5, 10 mM KCl, 1.5 mM MgCl_2_, 1 mM EDTA, 1 mM EGTA, 1 mM DTT, 250 mM sucrose, and protease inhibitors), and homogenised using a teflon homogeniser. The homogenate was centrifugated to remove cellular debris (2000 g/10 min). The supernatant was again centrifugated (12500 g/30 min) and 500 *μ*l solubilisation buffer (from caspase 9 colorimetric assay kit) was added to the supernatant representing the cytosolic fraction. The pellet was resuspended in 500 *μ*l buffer B (250 *μ*l buffer A plus 250 *μ*l solubilisation buffer) representing the mitochondrial fraction. Both fractions were subjected to cytochrome *c* ELISA assays according to the manufacturer's specifications. For each sample, the relative distribution of cytochrome *c* between cytosolic and mitochondrial fraction was calculated.

## RESULTS

### Ectopic expression of farnesylated Akt1 mediates chemoresistance in NCI H460 human lung cancer cells

To establish a cellular system that allows to analyse mechanisms of chemoresistance directly related to Akt, we stably transfected NCI H460 human NSCLC cells with an expression vector for constitutively active Akt. This plasmid encoded for Akt1 devoid of its N-terminal PH domain (replaced by a FLAG tag for antibody detection). To target Akt1 to the membrane for constitutive activation, we inserted a C-terminal sequence tag encoding a farnesylation motif and a stretch of basic amino acids (Schmidt *et al*, 2002).

**Figure 1:**
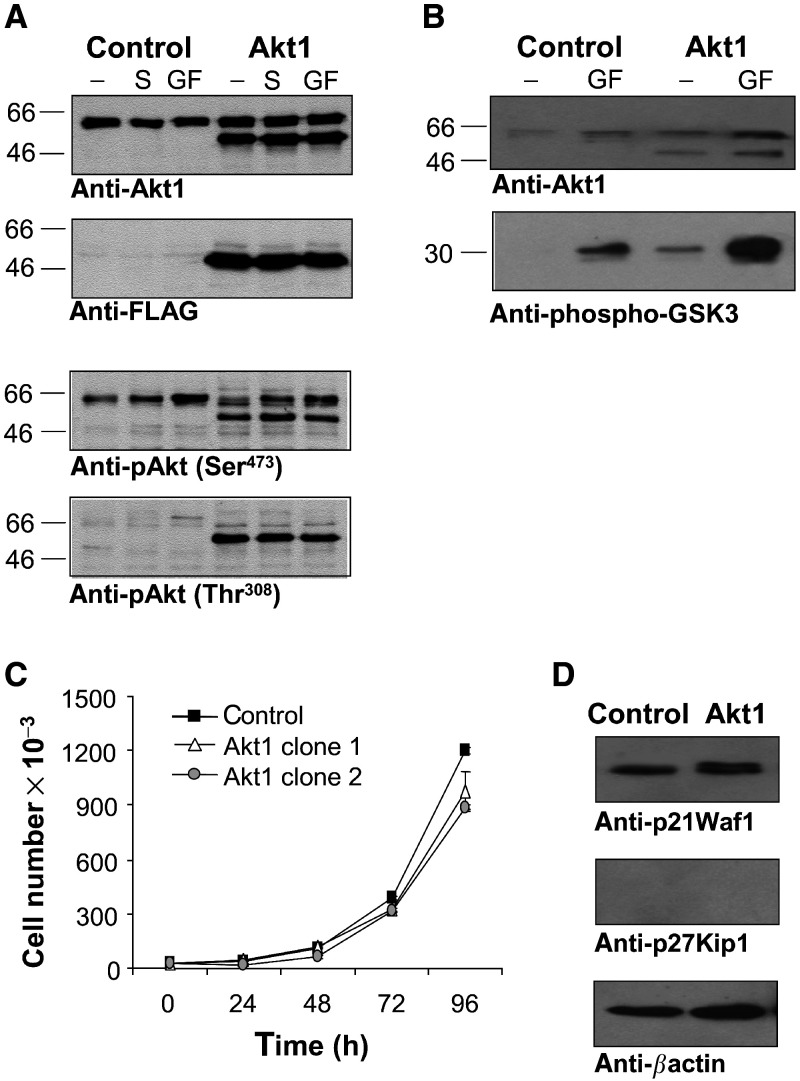
(**A**) Expression and activation of ectopically expressed farnesylated Akt1 in NCI H460 cells. Control transfected NCI H460 cells or cells expressing farnesylated Akt1 were serum starved overnight (-) and then stimulated for 15 min with medium containing 10% FCS (S) or 10% FCS plus a cocktail of 10 ng ml^−1^ each EGF, IGF-1, and PDGF (GF). Cell lysates were analysed by immunoblotting with antibodies specific for Akt1, FLAG-epitope, phospho-Akt (Thr 308) or phospho-Akt (Ser 473) as indicated. The ectopically expressed farnesylated Akt1 devoid of the PH domain migrates at an MW of approximately 50 kDa. (**B**) Akt kinase activity in NCI H460-Akt1 cells and in control cells. Cells were serum starved for 16 h and then stimulated as described above. Endogenous Akt1 from control transfected cells and endogenous Akt1 plus farnesylated Akt1 from NCI H460-Akt1 cells were immunoprecipitated with an Akt1 specific antibody. Immunoprecipitates were used in kinase assay reactions with GSK-3-fusion protein as substrate. Immunoblots of the reactions were assayed with antibodies specific for phospho-GSK (Ser 21/9) (lower panel) and Akt1 (upper panel). (**C**) Proliferation rates of NCI H460 control transfectants and NCI H460-Akt1 cells. 3 × 10^4^ cells (as indicated) per well were seeded into six wells into a medium containing 0.5% FCS. At the indicated time points, the cells were harvested by trypsinisation and cell numbers were determined using a Coulter counter. Each sample was determined in triplicates. Two independent NCI H460-Akt1 cell clones were analysed. (**D**) Expression analysis of p21^Waf1^ and p27^Kip1^ in NCI H460-Akt1 cells *vs* control cells. Cell lysates were analysed by immunoblotting with antibodies specific for p21^Waf1^, p27^Kip1^, and *β*-actin as a loading control.

Single-cell clones were analysed for ectopic expression of Akt1 by immunoblot analysis of cell lysates with an M2 anti-FLAG antibody, and positive clones (NCI H460-Akt1) were used in this study. To assess whether ectopically expressed farnesylated Akt1 was phosphorylated at threonine 308 and serine 473, selected clones were analysed in immunoblots for their phosphorylation status at these residues (Figure 1

). NCI H460-Akt1 cells were starved for 16 h and then stimulated with medium containing 10% serum or 10% serum plus growth factors (EGF, PDGF, and IGF-1). Cells were transfected with the empty vector were used as controls. The results are shown in Figure 1A: endogenous Akt1 was detected as a protein migrating at an *M*_r_ around 66 kDa, whereas ectopically expressed CA-Akt1 was detected with both, anti-Akt1 and anti-FLAG antibodies as a protein with an *M*_r_ of about 50 kDa (due to the lack of the PH domain). Phosphorylation of endogenous Akt1 at Ser473 was induced by stimulation of cells with a growth factor cocktail, and, to a lesser extent, by stimulation with serum alone, while phosphorylation at Thr308 was hardly detectable. In contrast, farnesylated Akt1 was phosphorylated at both residues irrespective of serum conditions or growth factor stimulation.

To determine the kinase activity of endogenous and ectopically expressed farnesylated Akt1, an *in vitro* kinase assay with immunoprecipitated Akt from cell lysates and GSK-3-fusion protein as substrate was performed under conditions as described for Figure 1A (for details, see Materials and methods). As shown in Figure 1B, endogenous Akt1 in control cells exhibited no detectable kinase activity after serum deprivation, but stimulation with serum and growth factors strongly induced kinase activity. In immunoprecipitates from NCI H460-Akt1 cells, kinase activity from ectopically expressed Akt1 could already be detected under serum-free conditions. Upon serum and growth factor stimulation, Akt kinase activity was induced to a much greater extent as in control cells. The results from the *in vitro* kinase assay and from the immunoblot analysis of Akt1 phosphorylation status show that ectopically expressed farnesylated Akt1 is constitutively activated in human NCI H460-Akt1 cells and that these cells display a higher Akt kinase activity than control cells irrespective of medium conditions. Surprisingly, we observed that CA-Akt1 transfected clones displayed a slight growth retardation compared to control transfected cells (Figure 1C). Analysis of the expression levels for the cell cycle inhibitors p21^Waf1^ and p27^Kip1^, respectively, revealed no changes in protein abundance. However, we observed a reduced electrophoretic mobility of p21^Waf1^ in NCI H460-Akt1 cells, which might be indicative of increased p21^Waf1^ phosphorylation (Figure 1D).

**Figure 2:**
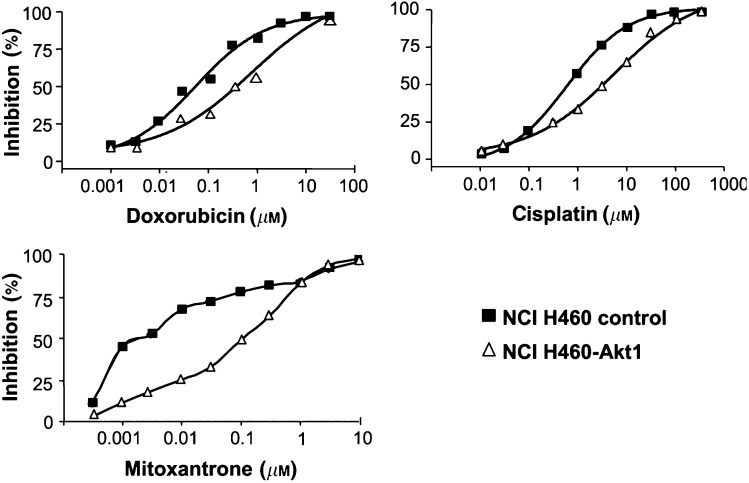
Decreased chemosensitivity of NCI H460-Akt1 cells towards doxorubicin, cisplatin, and Mitoxantrone. Control transfected NCI H460 cells (filled squares) or NCI H460-Akt1 cells (open triangles) were seeded in 96-well plates in medium containing 0.5% serum. After 24 h cells were treated with different concentrations of doxorubicin, cisplatin, or Mitoxantrone as indicated and incubated for another 72 h at 37°C. Cell viability was then determined with a standard XTT assay as described in ‘Materials and methods’. Values are the mean of at least three independent experiments.

To assess whether the ectopic expression of CA-Akt1 modulates the cellular response to treatment with chemotherapeutic regimen, we compared the sensitivity of control transfected human NCI H460 cells with the sensitivity of NCI H460-Akt1 cells towards a panel of chemotherapeutics, namely cisplatin, Mitoxantrone, 5-fluoruracil, doxorubicin, and paclitaxel. The viability of the cells after incubation with the substances for 72 h was determined with a standard XTT assay as described in Materials and methods. The most striking differences were observed with the DNA alkylating agents cisplatin and Mitoxantrone, and with the anthracycline doxorubicin: NCI H460-Akt1 cells displayed a 20-fold increased resistance towards the DNA alkylating agent Mitoxantrone as compared to control cells (IC_50_ 0.1 *vs* 0.005 *μ*M, respectively). An approximately 10-fold decreased sensitivity was observed upon treatment with the DNA alkylating agent cisplatin, whereas a 14-fold decrease in chemosensitivity was measured with the anthracycline doxorubicin (Figure 2

). Only small changes in chemosensitivity have been observed after incubation with the M-Phase specific tubulin inhibitor paclitaxel or the antimetabolite 5-fluorouracil (2.5- and 3-fold decrease, respectively) which primarily acts in the S-phase of the cell cycle. The IC_50_ values towards the various chemotherapeutics are summarised in Table 1

**Table 1:** Chemosensitivit y of control transfected NCI H460 cells or NCI H460-Akt1 cells

	**NCI H460**		
**Compound**	**IC_50_ (***μ***M) (Control)**	**IC_50_ (***μ***M) (Akt1)**	**Resistance factor**
5-FU	0.03	0.09	3
Cisplatin	0.6	5.6	9.3
Doxorubicin	0.055	0.78	14
Paclitaxel	0.002	0.005	2.5
Mitoxantrone	0.005	0.1	20

Values were determined as average from at least three independent experiments performed as quadruplicates. The resistance factor was calculated as the IC_50_ value of NCI H460-Akt1 cells divided by the IC_50_ value of control transfected NCI H460 cells.

. Importantly, the differential chemoresistance was only detected when cells were grown in low serum. If cells were cultured under normal medium conditions, the activation of endogenous Akt through serum components probably suffices to activate Akt-dependent antiapoptotic pathways. These data suggest that the expression of activated Akt1 in human NCI H460 NSCLC cells can desensitise this cell line especially towards DNA reactive chemotherapeutics, but – at least in our system – does hardly alter the sensitivity towards cell cycle specific agents such as paclitaxel.

### Modulation of apoptotic pathways by CA-Akt1 after chemotherapeutic treatment of NCI H460 lung cancer cells

Most of the chemotherapeutics kill tumour cells via the induction of apoptosis (Kaufmann and Earnshaw, 2000). To understand the molecular alterations that lead to decreased chemosensitivity of NCI H460-Akt1, we examined several apoptotic pathways and executors and focused our analyses on cisplatin- and Mitoxantrone-induced cell death, which were found to be most strongly influenced by CA-Akt1.

A very sensitive cell death ELISA detecting histone-associated DNA fragments was initially employed to determine the time points and concentrations of chemotherapeutics at which apoptosis is already initiated in control cells but not yet visible by DNA fragmentation in CA-Akt1-transfected cells. The optimal time points and concentrations were found to be 20 *μ*M cisplatin (incubation time 36 h) and doses between 150 and 400 nM Mitoxantrone (incubation time 24 h) (data not shown). Further experiments were conducted using these compounds at the indicated concentrations.

The activation of certain caspases is indicative of specific pathways that are activated in order to execute apoptotic stimuli (e.g. death receptor-mediated activation of caspases 8 and 10 or activation of caspase 9 by the apoptosome (Peter and Krammer, 1998; Zou *et al*, 1999). Upon induction of caspase cascades, a subset of cellular caspase downstream target proteins are proteolytically cleaved. Poly-ADP-ribose polymerase (PARP), one such target protein, is one of the earliest proteins targeted for a specific cleavage to a 89-kDa C-terminal fragment with a reduced catalytic activity and a 24 kDa N-terminal peptide, which retains the DNA-binding domains during the initiation of apoptosis (Peter and Krammer, 1998).

For the initial examination of caspase activation in Akt-mediated chemoresistance, we treated NCI H460-Akt1 cells and control cells with 20 *μ*M cisplatin for 36 h. Subsequently, immunoblots with antibodies specific for proteolytic cleavage of initiator and effector caspases as well as the DNA repair enzyme PARP were performed.

**Figure 3:**
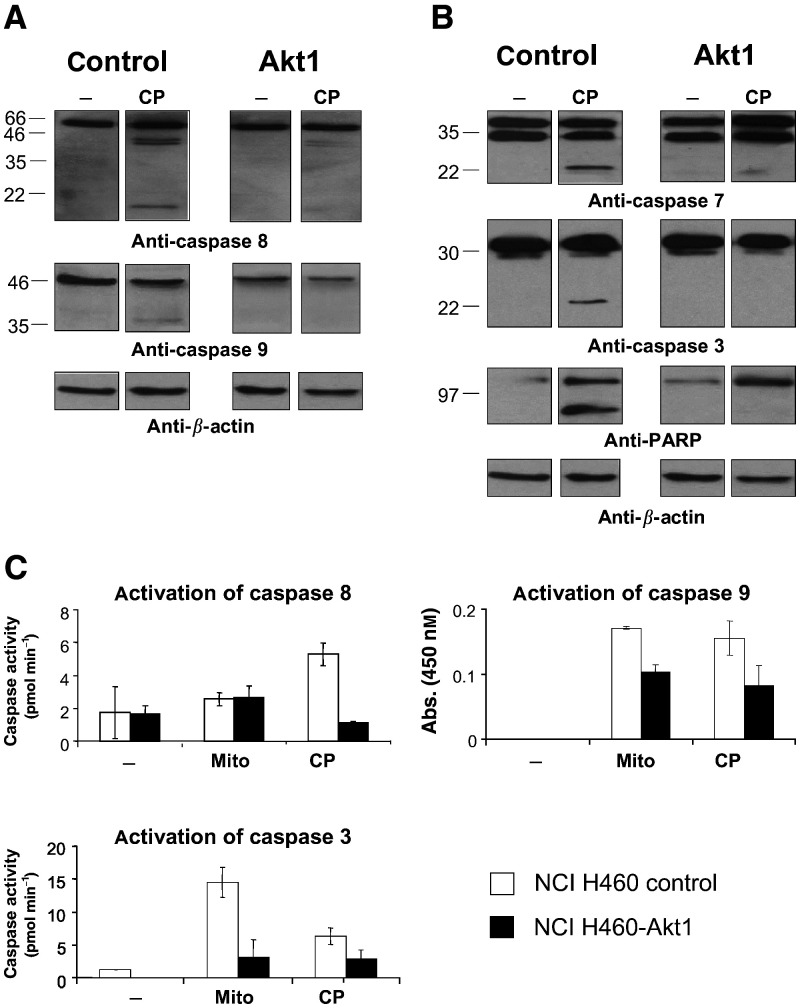
(**A**) Activation of initiator caspases after treatment of NCI H460 cells with cisplatin. Control transfected NCI H460 cells or NCI H460-Akt1 cells were grown in medium containing 0.5% serum overnight and afterwards treated with cisplatin (20 *μ*M) for 36 h. Cell lysates were analysed by immunoblotting with antibodies specific for caspase 8 or caspase 9 as indicated. (**B**) Activation of effector caspases and PARP after treatment of NCI H460 cells with cisplatin. Lysates from cells treated as described above were prepared and analysed by immunoblotting with antibodies specific for caspase 3, caspase 7, and PARP as indicated. (**C**) Enzymatic assays of caspase activity. Cells were treated as described above. Additionally, samples from cells treated with 400 nM Mitoxantrone for 24 h were included. Caspase 3, 8, and 9 activities from lysates were determined by a colorimetric assay as described in ‘Materials and methods’.

Control cells treated with cisplatin showed activation of initiator caspases 8 and 9, effector caspases 3 and 7 as well as the caspase target PARP (Figure 3A and B

, left panels). In contrast, cleavage of caspases 8, 9, and 7 in NCI H460-Akt1 cells was greatly diminished, while no cleavage of caspase 3 and PARP was observed at these time points (Figure 3A and B, right panels). Overall, caspase activation upon treatment with Mitoxantrone (150 nM for 24 h) was much weaker as compared to treatment with cisplatin (data not shown).

Colorimetric caspase activity assays were conducted in order to further substantiate the observation of differential caspase activation in CA-Akt1 transfected NCI H460 cells. After incubation with either Mitoxantrone (400 nM; 24 h incubation time) or cisplatin (20 *μ*M; 36 h incubation time), lysates from control cells exhibited a significantly stronger caspase 3 and 9 activity compared to cells expressing constitutively activated Akt1 (Figure 3C). Caspase 8 activity was induced to 5.3 pmol min^−1^ in control cells after cisplatin treatment compared to 1.4 pmol min^−1^ in untreated cells. No increase in caspase 8 activity was detected for NCI H460-Akt1 cells incubated with cisplatin. Treatment with Mitoxantrone led to a slight but statistically not significant induction of caspase 8 activity in both control and NCI H460-Akt1 cells. Taken together, activation of both, initiator and effector caspases, is quantitatively reduced and kinetically delayed in NCI H460-Akt1 cells. However, no general qualitative differences were observed, indicating that ectopic expression of CA-Akt1 is only capable of delaying certain apoptotic pathways, but not of completely suppressing the activity of specified caspases.

**Figure 4:**
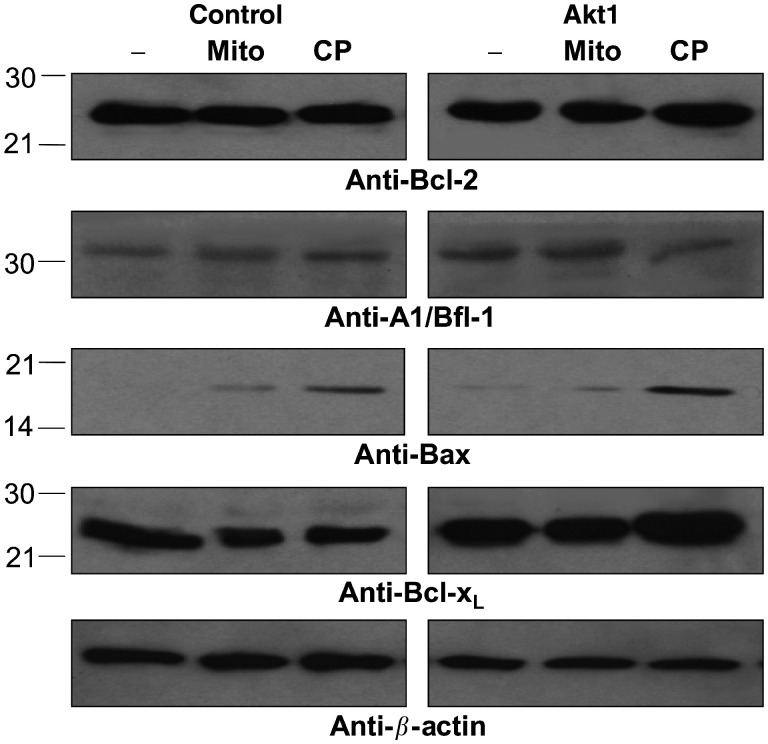
Expression analysis of Bcl-2 family proteins in NCI H460 cells. Control transfected NCI H460 cells or NCI H460-Akt1 cells were grown in medium containing 0.5% serum overnight (-) and afterwards treated with 150 nM Mitoxantrone (Mito) for 24 h or 20 *μ*M cisplatin (CP) for 36 h, respectively. Equal amounts of cell lysates were analysed by immunoblotting with antibodies specific for Bcl-2, A1/Bfl-1, Bax, and Bcl-x_L_ as indicated.

It has been described that activated Akt1 can influence the onset of apoptosis by interfering with proteins from the mitochondrial pathway of apoptosis (Datta *et al*, 1997; Pugazhenthi *et al*, 2000). Furthermore, the induction of apoptosis by chemotherapy in NCI H460 cells has been shown to be dependent on a functional mitochondrial pathway (Ferreira *et al*, 2000). The initial step is the release of cytochrome *c* from the mitochondria by proteins of the Bcl-2 family. To exploit the potential chemoprotective role of Bcl-2 family proteins in NCI H460-Akt1 cells, the expression of Bcl-2, Bfl-1, Bcl-x_L_, Bax, and Bcl-x_s_ was investigated after exposure to Mitoxantrone or cisplatin as described above (Figure 4

). The expression of Bcl-2 and Bfl-1 proteins was unchanged in NCI H460-Akt1 cells *vs* control cells, regardless of chemotherapeutic treatment, while Bax expression was induced by Mitoxantrone and cisplatin to a similar extent in both cell transfectants. The expression of Bcl-x_s_ was barely detectable (data not shown). Most notably, Bcl-x_L_ protein levels were increased in NCI H460-Akt1 cells compared to control cells, which might account for an Akt1-mediated antiapoptotic mechanism.

**Figure 5:**
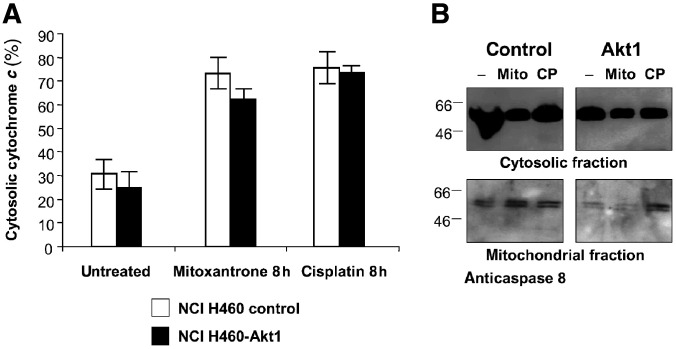
Cytochrome *c* release from mitochondria after treatment of NCI H460 cells with Mitoxantrone or cisplatin. Control transfected NCI H460 cells or NCI H460-Akt1 cells were grown in medium containing 0.5% serum overnight (-) and treated with 400 nM Mitoxantrone (Mito) or 20 *μ*M cisplatin (CP) for 8 h, respectively. (**A**) Cell lysates were fractionally centrifugated and analysed with a cytochrome *c* ELISA as described in ‘Materials and methods’. Each value was determined as triplicate. (**B**) Aliquots of cell lysates were analysed by Western blotting with an antibody specific for caspase 8 as a control for cellular fractionation.

For further analysis of the mitochondrial apoptotic pathway, we determined the release of cytochrome *c* from mitochondria. Therefore, lysates from control and NCI H460-Akt1 cells treated with 400 nM Mitoxantrone or 20 *μ*M cisplatin for 8 h, respectively, were fractionally centrifuged. Cytosolic and mitochondrial concentrations of cytochrome *c* were determined by ELISA as described in ‘Materials and methods’. As shown in Figure 5

, incubation with chemotherapeutics induced cytochrome *c* release from mitochondria into the cytosol, thereby increasing the cytosolic concentration of cytochrome *c* from approximately 30 to 75%. No differences were observed between control and NCI H460-Akt1 cells, indicating that activated Akt1 did not prevent the release of cytochrome *c* from the mitochondria. Caspase 9 may be phosphorylated and thereby inactivated by Akt upon phosphorylation at serine 198 (Cardone *et al*, 1998). However, we only observed very subtle differences in the caspase 9 phosphorylation (data not shown), making selective caspase 9 inactivation unlikely to be causative for Akt1-mediated chemoresistance.

**Figure 6:**
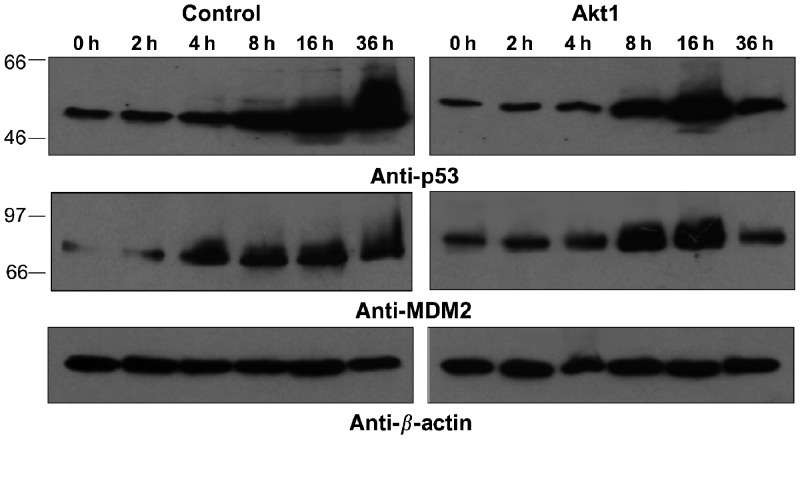
Induction of p53 in Akt1-transfected NCI H460 cells. Control transfected NCI H460 cells or NCI H460-Akt1 cells as indicated were grown in medium containing 0.5% serum and treated with cisplatin (20 *μ*M) for 2, 4, 8, 16, and 36 h, respectively. At each time point, cell lysates were generated and analysed by immunoblotting with antibodies specific for p53, MDM2, and *β*-actin.

Another important pathway that is instrumental for the response of tumour cells towards chemotherapeutic agents includes the activation of the p53 pathway. Activation of p53 occurs via multiple serine phosphorylations by serine/threonine kinases such as ATM, ATR, Chk2 or DNA-PK, leading to stabilisation, activation, and translocation into the nucleus, where p53 acts as transcription factor interfering with transcription of multiple genes involved in cell cycle regulation, DNA repair, and apoptosis (Sionov and Haupt, 1999). To investigate whether constitutive activation of Akt1 interferes with the activation of p53, control cells and NCI H460-Akt1 cells were treated with 20 *μ*M cisplatin for 2, 4, 8, 16, and 36 h, respectively. Lysates were analysed with specific antibodies for expression levels of p53 and the p53 antagonist MDM2. The results are summarised in Figure 6

: NCI H460-Akt1 cells showed a similar kinetics of p53 upregulation as compared to control cells. A delayed onset of upregulation, however, was observed for MDM2, which was induced as early as 4 h of cisplatin exposure in control transfected cells and remained stable for at least 36 h. MDM2 upregulation was observed only at 8 h of cisplatin exposure in NCI H460-Akt1 cells and declined already after 16 h. Very similar results of delayed MDM2 upregulation in NCI H460-Akt1 *vs* control cells were observed when cells were treated with 400 nM Mitoxantrone for time points between 2 and 24 h (data not shown).

From our data, we conclude that the expression of constitutively activated Akt1 in NCI H460 human NSCLC cells can cause partial resistance towards a class of chemotherapeutics, most pronouncedly towards drugs interacting with DNA. Molecular pathway analyses revealed that CA-Akt increases the apoptotic threshold or time course of onset of multiple apoptotic pathways. However, the sole ectopic expression of a constitutively active form of Akt1 does not suffice to completely suppress the chemotherapy-induced activation of defined apoptotic triggers.

## DISCUSSION

PKB/Akt has been described as a central mediator of antiapoptotic signalling and as a mediator of reduced sensitivity of cancer cells towards chemotherapeutic agents (West *et al*, 2002). Recently, we could show that expression of constitutively active farnesylated Akt1 (CA-Akt) renders MCF10A mammary epithelial cells and A549 non-small-cell lung cancer (NSCLC) cells more resistant to the treatment with cell cycle independent chemotherapeutics such as cisplatin and Mitoxantrone (Schmidt *et al*, 2002). In this study, we examined the effects of CA-Akt expression in human NCI H460 NSCLC cells regarding chemosensitivity and the modulation of apoptotic pathways. Utilising an expression vector encoding Akt1 devoid of its PH domain and a C-terminally fusion of a farnesylation sequence described recently (Schmidt *et al*, 2002), we generated stable NCI H460 cell clones expressing CA-Akt. This consititutive activation of PKB/Akt resulted in a decreased sensitivity of NCI H460 cells towards a panel of chemotherapeutic agents currently used in the clinic (Table 1). It has to be noted that we observed the mediation of chemoresistance by CA-Akt primarily under conditions of low serum and to a far lesser extent in full growth medium. We explain this observation by the fact that endogenous Akt can be activated by serum components (Figure 1). Under these conditions, the activity of ectopically expressed CA-Akt may not provide a further survival advantage compared to active endogenous Akt alone. The most profound desensitisation was detected for Mitoxantrone, doxorubicin, and cisplatin (Figure 2). These agents can exert their cytotoxic mode of action by inducing DNA damage irrespective of the cell cycle phase. Mitoxantrone and doxorubicin have been described to intercalate with DNA at high concentrations, but also to inhibit Topoisomerase II, thereby leading to persistant double-strand breaks of DNA (Bodley *et al*, 1989; Boland *et al*, 2000). Cisplatin has been shown to alkylate DNA and to lead to the activation of caspase 8 with subsequent initiation of a caspase cascade (Ferreira *et al*, 2000). Surprisingly, no significant modulation of chemosensitivity was observed towards cell cycle specific drugs such as paclitaxel. Thus, we can exclude that the chemoresistance in NCI H460-Akt1 cells is simply due to the reduced growth rate of the transfectants.

To further study the molecular mechanisms underlying the CA-Akt-mediated altered chemosensitivity, we analysed various apoptotic pathways. In summary, quantitative or kinetic differences were observed in the suppression or delay of molecular pathways of apoptosis induction, but no complete inhibition could be accounted to CA-Akt1. Different pathways for the induction of apoptosis have been discovered, for example, the activation of death receptors or the release of cytochrome *c* from the mitochondria. Both pathways comprise the activation of caspase cascades, leading to cell death. The proteolytic cascade consists of initiator caspases such as caspases 8, 9, and 10, and downstream effector caspases, for example, caspase 3 and 7 (Nunez *et al*, 1998). Akt1 has been described to suppress apoptotic stimuli by interfering with apoptotic pathways, for example, by phosphorylation of caspase 9 (Cardone *et al*, 1998) or the proapoptotic Bcl-2 homologue Bad (Datta *et al*, 1997). Analysing the activation of caspases upon stimulation with Mitoxantrone or cisplatin at concentrations and time points leading to DNA fragmentation in control cells but not in NCI H460-Akt1 cells exhibited a kinetic delay as well as quantitative, but not qualitative differences in the activation of the caspase cascade. Western Blot analysis revealed that upon treatment with cisplatin control cells showed cleavage of both initiator caspases 8 and 9 as well as the effector caspases 7 and 3 and the caspase 3/7 target protein PARP, while only caspase 8 was weakly activated in NCI H460-Akt1 cells at that time point (Figure 3A and B). These findings were supported by caspase activity assays showing a profound reduction in the activation of initiator caspases 8 and 9 as well as the effector caspase 3 in NCI H460-Akt1 cells compared to control cells upon treatment with cisplatin, while upon treatment with Mitoxantrone an altered activation was only observed in caspase 9 and 3 as caspase 8 is hardly activated by Mitoxantrone at all (Figure 3C).

The mitochondrial apoptosis pathway plays an important role in the execution of chemotherapy-induced apoptosis. Cytosolic cytochrome *c* binds to Apaf-1 and together with caspase 9 forms a complex termed ‘apoptosome’. Within the ‘apoptosome’, caspase 9 is proteolytically cleaved and activated (Cain *et al*, 2000). Yet, neither differences in the release of cytochrome *c* from mitochondria (Figure 5) nor the expression of the ‘apoptosome’ associated protein Apaf-1 (data not shown) were detected between CA-Akt and control cells upon treatment with cisplatin or Mitoxantrone. Only very subtle differences in the caspase 9 phosphorylation, which is known to be inactivated by Akt1 (Cardone *et al*, 1998), could be observed in NCI H460-Akt1 cells (data not shown), making selective caspase 9 inactivation unlikely to be causative for Akt1-mediated chemoresistance. Other proteins involved in mitochondrial membrane integrity are members of the Bcl-2 family. One potential mechanism by which Akt1 may interfere with proapoptotic members of this family is the inhibition of a Bax conformational change preventing disruption of mitochondrial inner membrane potential and caspase 3 activation (Yamaguchi and Wang, 2001). Analysis of the expression levels of Bcl-2 and Bfl-1 or the induction of Bax expression after exposure to Mitoxantrone or cisplatin did not reveal differences between NCI H460-Akt1 and control cells. In contrast, protein levels of antiapoptotic Bcl-x_L_ were increased in NCI H460-Akt1 cells compared to control cells. Bcl-x_L_ overexpression has been shown to protect cells from p53-mediated apoptosis (Schott *et al*, 1995). Thus, the induction of Bcl-x_L_ overexpression might account for an Akt1-mediated antiapoptotic mechanism.

The p53 tumour-suppressor protein pathway also plays an important role in the transduction of proapoptotic signals towards various forms of cellular stress, such as DNA damage. Upon DNA damage, p53 is activated via phosphorylation by Chk1, Chk2, ATM, and ATR, and may translocate into the nucleus, where it induces the transcription of pro-apoptotic proteins such as Bax, as well as its antagonist MDM2 as a negative feedback loop (Sionov and Haupt, 1999). PKB/Akt has been shown to phosphorylate MDM2 at Ser 166 and Ser 186 (Mayo and Donner, 2001). After phosphorylation, MDM2 may likewise translocate from the cytoplasm into the nucleus, where it can block p53 transactivation and promote p53 degradation. Our results show an induction of p53 and successively MDM2 upon treatment with cisplatin (Figure 6). However, induction of MDM2 expression as a p53-regulated gene was delayed in CA-Akt-expressing cells compared to control cells. This may suggest that the onset of p53 transactivation itself is delayed by the expression of CA-Akt. Moreover, expression of MDM2 and p53 declined after 36 h in NCI H460-Akt1 cells, but no decline was detected in control cells.

In summary, our results show that ectopic expression of constitutively activated Akt1 in NCI H460 human NSCLC cells can cause partial resistance towards a class of chemotherapeutics and may increase the apoptotic threshold or delay time course of certain apoptotic pathways, but does not completely suppress the activity of specified caspases or the chemotherapy-induced apoptosis. It is interesting to note that chemoresistance is only conferred towards non-cell cycle-dependent chemotherapeutics primarily interfering with DNA. From this point of view, it will be worthwhile to study potential target proteins of Akt directly involved in the sensing of DNA damage such as Chk1 and 2, ATM, ATR, and other proteins involved in the DNA damage checkpoint pathway. Furthermore, our data support the notion that a pharmaceutical drug-inhibiting Akt activity may not be sufficient to kill cancer cells on its own, but may be used to lower the apoptotic threshold and to resensitise chemoresistant tumours in combination treatment with defined chemotherapeutic drugs.
